# Broviac Catheter–Related Aortic Valve Infective Endocarditis Complicated With Massive Aortic Regurgitation Requiring Emergency Surgery: A Case Report

**DOI:** 10.1155/2024/1093820

**Published:** 2024-10-24

**Authors:** Małgorzata Wilawer, Waldemar Elikowski, Natalia Fertała, Arkadiusz Włodarski, Patryk Szczęśniewski, Paulina Anna Ratajska, Paweł Bugajski

**Affiliations:** ^1^Department of Internal Medicine, Józef Struś Hospital, Poznań, Poland; ^2^Intensive Care Unit (ICU), Józef Struś Hospital, Poznań, Poland; ^3^Department of Cardiac Surgery, Józef Struś Hospital, Poznan, Poland

**Keywords:** Broviac catheter, case report, echocardiography, infective endocarditis, *Staphylococcus epidermidis*

## Abstract

**Introduction:** Broviac catheter is a type of central venous catheter (CVC) used for long-term parenteral nutrition in specific patients, e.g., diagnosed with intestinal failure as short bowel syndrome (SBS). The way of the catheter insertion is conceived to minimalize the risk of infections. However, CVC-related blood stream infections (CVC-BSIs), including infective endocarditis (IE), remain most important complications associated with Broviac catheter. *Staphylococcus epidermidis* stands out as a prevalent pathogen. The increasing number of CVCs results in an increased incidence of healthcare-associated IE. Complete parenteral treatment is an independent risk that increases the likelihood of IE. Treatment of IE is mainly based on antibiotic therapy, but in certain cases, surgical treatment is needed.

**Presentation of Case:** A 71-year-old female with SBS who had been receiving total parenteral nutrition through the Broviac catheter for several months was admitted in a serious condition with significant weakness, increasing shortness of breath, deteriorating cough, fever, low blood pressure, and heart palpitations. Echocardiography revealed severe aortic valve IE with a large, longitudinal, highly mobile vegetation (up to 40 mm) and massive aortic regurgitation with pulmonary edema. Fast pathogen detection in the patients' blood (*S. epidermidis*) was obtained using PCR-based multiplex test. Due to life-threatening conditions, emergency surgery with aortic valve replacement was performed. Consistent rehabilitation resulted in good condition achievement. Follow-up echocardiography showed normal function of the aortic valve bioprosthesis.

**Conclusion:** The use of CVC, including Broviac catheter, is associated with an increased risk of infections, including IE. Treatment-resistant severe HF complicating IE requires emergency surgery.

## 1. Introduction

Broviac catheter is a type of central venous catheter (CVC) inserted into the superior vena cava or right atrium by way of the cephalic vein or external jugular vein used for total home parenteral nutrition [1, 2]. This approach provides a practical way to allow long-term nutritional support of specific patients, e.g., diagnosed with intestinal failure as short bowel syndrome (SBS) [3]. Broviac and similar catheters (Hickman, Groshong) are tunneled under the chest skin before entering a large vein, which should result in a low infection rate. However, CVC-related blood stream infections (CVC-BSIs), including infective endocarditis (IE), remain most important complications associated with these catheters [4, 5]. CVC-BSI is a serious clinical problem whose frequency is probably underestimated [6]. The most common microorganisms causing Broviac catheter-related infections are *Staphylococcus epidermidis*, *Staphylococcus aureus*, and *Candida albicans* [7]. Coagulase-negative Staphylococci (CoNS) are responsible for 13%–38% of nosocomial IE and tend to have an insidious clinical course. Left-sided IE associated with CVC-BSI is observed in 33% of the cases [8, 9]. According to 2023 European Society of Cardiology Guidelines for the management of endocarditis, surgical timing in a patient with left-sided IE includes severe heart failure (HF) manifesting as cardiogenic shock or pulmonary edema, uncontrolled infection, and high risk of embolism or established embolism. The first condition indicates the need of emergency surgery (within 24 h) and the remaining two suggest urgent surgery (within 3–5 days) [8].

## 2. Case Report

**Figure 1:**
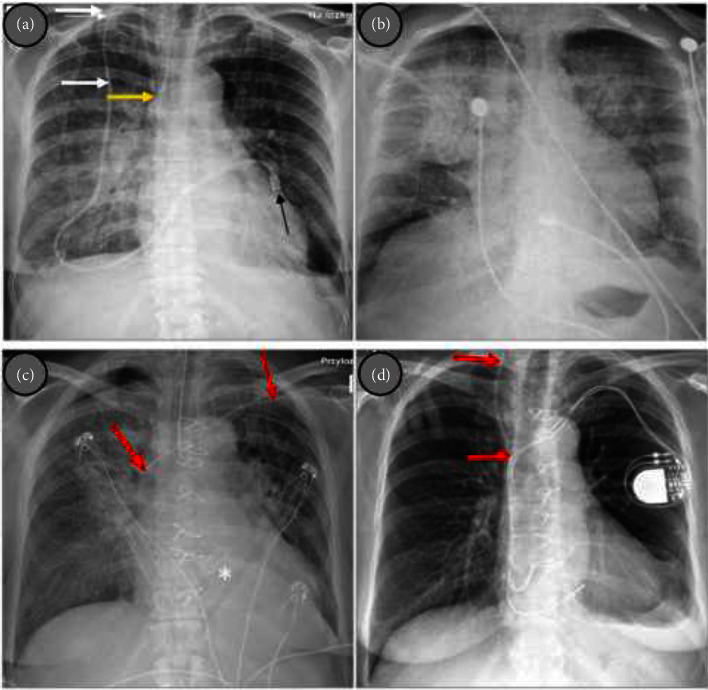
Chest x-ray: pulmonary congestion and bilateral pleural effusion; white arrows: tunneled part of the Broviac catheter, yellow arrow: catheter in the superior vena cava—SVC, black arrow: catheter's cap (a), few hours later, after Broviac catheter removal; see progression of the pulmonary congestion to pulmonary edema (b), the day after aortic valve replacement with bioprosthesis—AVB—white asterix (c), and after pacemaker implantation (d). Red arrows—new (percutaneous) central venous catheter inserted into the SVC directly through the skin via left subclavian vein and then via right internal jugular vein.

**Figure 2:**
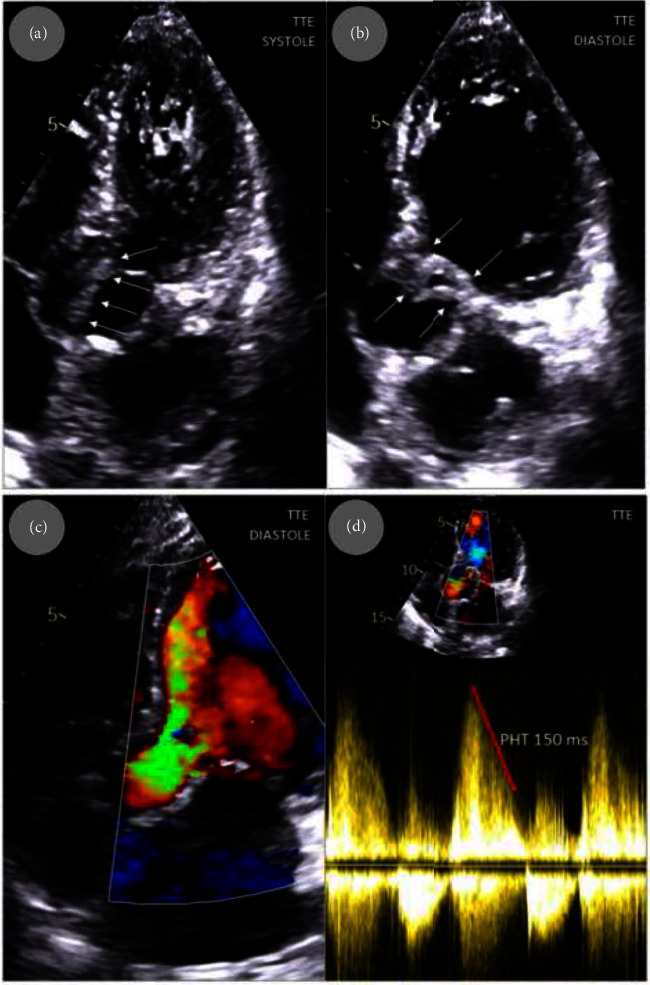
Echocardiography at admission: large, longitudinal, highly mobile vegetation (white arrows) on aortic valve moving in systole into aortic root (a) and presenting as rolled up structure in diastole (b); massive aortic regurgitation in color Doppler imaging (c); and in spectral Doppler imaging—see very short pressure half time (red arrow) (d). TTE, transthoracic echocardiography, PHT, pressure half time.

A 71-year-old white female who had been receiving total parenteral nutrition through the Broviac catheter for several months was admitted in serious condition with significant weakness, increasing shortness of breath, deteriorating cough, fever, low blood pressure, and heart palpitations. The patient's medical history contained anemia, SBS after right hemicolectomy for nonmetastatic colon cancer, laparotomy due to adhesive obstruction of the small intestine, and a two-site resection of the small intestine with anastomotic ileostomy. The patient had no prior history of antibiotic use before hospitalization. On admission, she was hemodynamically unstable with hypotension (and especially low diastolic pressure). Heart auscultation revealed tachycardia and diastolic murmur suggestive of aortic regurgitation while lung examination was typical for severe pulmonary congestion which was confirmed in chest x-ray (Figure 1(a)). Laboratory tests showed normocytic anemia (hemoglobin 4.2 mmol/L), leukocytosis (14.5 thousand/*μ*L), hypoalbuminemia (25 g/L), increased creatinine levels (161 *μ*mol/L), and raised inflammatory markers: CRP and procalcitonin (110.4 mg/dL and 2.34 ng/mL, respectively), as well as elevated troponin I and NT-proBNP levels (1272 ng/L and 33,979-pg/mL, respectively). Transthoracic echocardiogram (TTE) revealed large longitudinal, highly mobile vegetation (up to 40 mm) on the aortic valve and severe aortic valve regurgitation (Figure 2). The implementation of a multiplex PCR test facilitated the rapid detection of pathogen DNA in the blood, identifying *S. epidermidis* as the only organism among the 33 pathogens detectable by the assay. Antibiotic therapy with gentamycin (160 mg once a day) and vancomycin (1000 mg twice a day) was started. The Broviac catheter was removed as a potential source of infection; later microbiological workup results confirmed *S. epidermidis* as a pathogen of the catheter infection. Due to life-threatening conditions, the patient required emergency surgery especially since her state was rapidly deteriorating and control chest x-ray showed a progression of congestion to pulmonary edema (Figure 1(b)).

**Figure 3:**
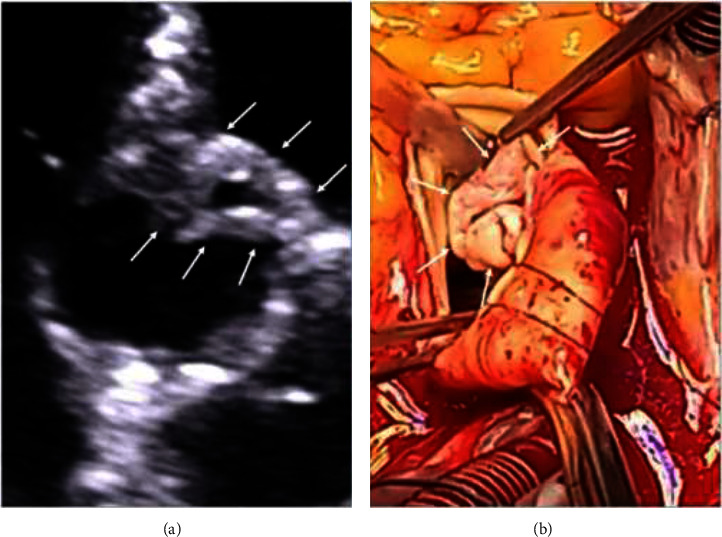
Large aortic valve vegetation found during surgery (a) and image comparison with echocardiographic presentation (b). The white arrows indicate vegetation.

**Figure 4:**
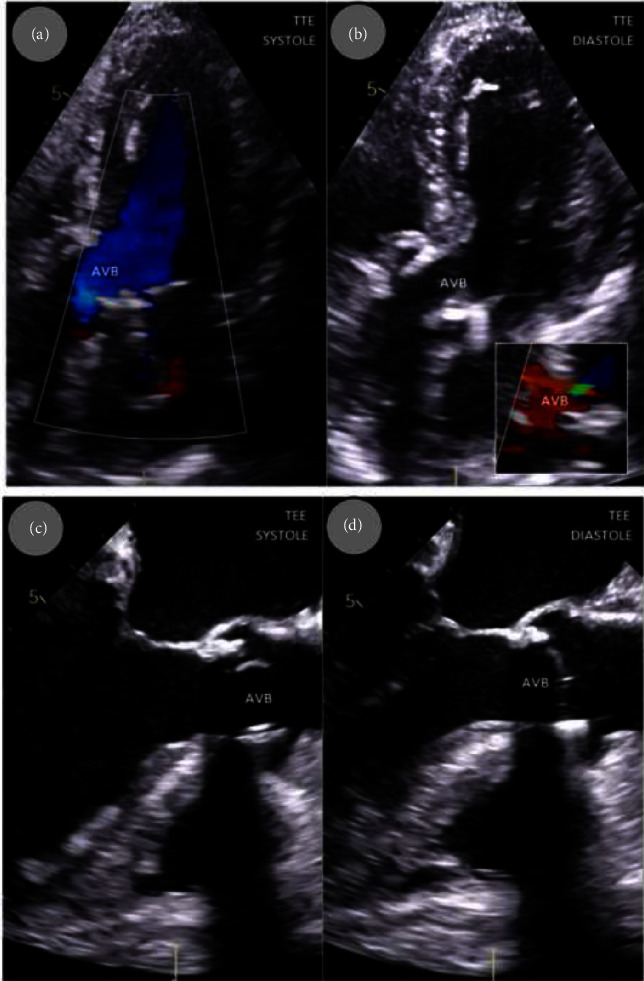
Echocardiography after aortic valve bioprosthesis implantation: normal color flow during systole (a), trace aortic regurgitation seen in diastole—zoom in lower/right site (b); normal aortic valve bioprosthesis (AVB) without vegetation seen in transesophageal echocardiography in systole (c); and diastole (d). TEE, transesophageal echocardiography.

After few hours' stay in intensive care unit (ICU), she was transferred to Cardiac Surgery Department where aortic valve replacement (AVR) with the Carpentier–Edwards Perimount Magna 21A pericardial bioprosthesis was performed (Figure 1(c)). The size of vegetation on the aortic valve was found to be bigger that in echocardiography examination (Figure 3). Complete destruction of the valve explained massive aortic regurgitation as a cause of pulmonary edema. Despite the technically successful intervention, the patient remained in a serious condition associated with respiratory and renal failure which resulted in prolonged (1 month) stay in the ICU. Mechanical ventilation was performed for 3 weeks, and antibiotic therapy was continued including broad-spectrum antibiotics due to concomitant infections. Before she returned to the Department of Internal Medicine, she had developed complete heart block and underwent dual-chamber pacemaker implantation (Figure 1(d)); blood cultures were negative at the time. Consistent rehabilitation allowed for a slow, gradual improvement in her physical and mental condition as she had been bedridden for almost for 2 months, taking into account prehospital period. Follow-up echocardiography showed normal function of the aortic valve prosthesis (Figure 4). Inflammatory markers and renal function tests returned to normal values (CRP: 12.1 mg/L, procalcitonin: 0.22 ng/mL, and creatinine: 81 *μ*mol/L). There were no signs of cancer recurrence. She was qualified to continue parenteral nutrition and for an intestinal reconstruction surgery.

## 3. Discussion

CVCs have become a lifesaver for many severely ill patients, but their use exposes patients to the risk of local and systemic infections such as BSI. More than 60% of all nosocomial infections are catheter related and most of them are CVC associated. In other words, the incidence of healthcare-associated IE is on the rise, mainly due to the increased use of CVC [6, 8]. Risk of CVC-BSI is compounded by factors such as the catheter type, insertion site, healthcare team expertise, timing of catheter placement, further manipulations, and the patient's clinical status. Total parenteral nutrition is an independent risk factor. The primary evidence-based strategies for reducing CVC-BSI include sterile catheter insertion, meticulous hand hygiene, the use of sterile surgical gloves for any catheter manipulation, the use of 2% chlorhexidine with adequate drying time before insertion, avoidance of the femoral site for catheter placement, and timely removal of unnecessary catheters [10]. The most common source of CVC-related infections are microorganisms that colonize the catheter sockets and the skin surrounding the catheter insertion site, which is why approximately 65% of those infections originate from the skin flora. CoNS adhere very easily to polymer surfaces and some strains can produce an extracellular polysaccharide called “slime” that acts as a barrier for multinucleated cells [11].

Another highly plausible mechanism for IE in individuals with CVC is the occurrence of endothelial damage resulting from forceful injections into the catheter, subsequently inducing intra-atrial flow. While most of the patients with catheter-related IE typically manifest with right-sided vegetation, in our patient, left-sided vegetation on the aortic valve was found. This observation could suggest the presence of underlying valvular conditions or a patent foramen ovale, but such diagnoses were not confirmed [12, 13].

*S. epidermidis* is a typical Gram + bacterium that occurs on the surface of the skin and mucous membranes, considered as a commensal organism belonging to the CoNS. It is a common and important nosocomial pathogen that produces biofilm and colonizes implanted medical devices such as catheters, and pacemaker leads or prosthetic valves, which may cause subacute or chronic bacteremia. However, it should be confirmed that the culture is positive due to the actual infection with *S. epidermidis* being the pathogen and not due to contamination of the sample [14]. PCR-based multiplex tests are probably superior (faster and more accurate) compared to conventional blood cultures for identification of BSI causative pathogens [15].

The primary mode of IE treatment involves antibiotics, although certain situations may warrant surgical intervention so current guidelines for the management of IE underlies the need for collaboration of multidisciplinary endocarditis team of specialists. Therefore, early cardiac surgery consultation is recommended to assess the best therapeutic approach at an early stage [8]. Emergency surgical indications encompass endocarditis affecting the mitral valve or aortic valve with significant regurgitation, valve flow obstruction, or a fistula, leading to treatment-resistant pulmonary edema or cardiogenic shock [8]. Moreover, in cases of left-sided IE, the prognosis is unfavorable when neurological complications occur; then, the mortality rate reaches 20%–30%. This rate has remained relatively constant over several decades and urgent surgery, when a patient has large valvular vegetations, should counteract this problem [8, 16].

Permanent complete heart block occurs in 3%–6% of the patients undergoing AVR and IE as the indication for surgery was found be one of its predictors [17]. Immediate epicardial pacemaker implantation should be considered in patients undergoing surgery for valvular IE if one of the following predictors of persistent atrioventricular block is present: preoperative conduction abnormality, *S. aureus* infection, aortic root abscess, tricuspid valve involvement, or previous valvular surgery. Unfortunately, there were no such predictors in our patient and temporary perioperative pacemaker was removed after a week [18].

In a patient with a history of CVC-related IE, intravenous pacemaker implantation as well as new CVC insertion may be associated with an increased risk of IE relapse. However, in our patient, long-term postsurgical antibiotic therapy could prevent this complication.

## 4. Conclusions

Broviac catheter as well as similar CVCs' use is associated with an increased risk of IE. The course of the infection may be insidious. Infected CVC should be immediately removed. Treatment-resistant severe HF requires emergency cardiac surgery. Permanent complete heart block is a rare complication of AVR.

## Data Availability

Data sharing is not relevant to this article since no datasets were created or examined in the present study.
